# Utilization of Microemulsions from *Rhinacanthus nasutus* (L.) Kurz to Improve Carotenoid Bioavailability

**DOI:** 10.1038/srep25426

**Published:** 2016-05-06

**Authors:** Nai-Hsing Ho, Baskaran Stephen Inbaraj, Bing-Huei Chen

**Affiliations:** 1 Department of Food Science, Fu Jen University, Taipei 242, Taiwan

## Abstract

Carotenoids have been known to reduce the risk of several diseases including cancer and cardiovascular. However, carotenoids are unstable and susceptible to degradation. *Rhinacanthus nasutus* (L.) Kurz (*R. nasutus*), a Chinese medicinal herb rich in carotenoids, was reported to possess vital biological activities such as anti-cancer. This study intends to isolate carotenoids from *R. nasutus* by column chromatography, identify and quantify by HPLC-MS, and prepare carotenoid microemulsions for determination of absolute bioavailability in rats. Initially, carotenoid fraction was isolated using 250 mL ethyl acetate poured into an open-column packed with magnesium oxide-diatomaceous earth (1:3, w/w). Fourteen carotenoids including internal standard β-apo-8′-carotenal were resolved within 62 min by a YMC C30 column and gradient mobile phase of methanol-acetonitrile-water (82:14:4, v/v/v) and methylene chloride. Highly stable carotenoid microemulsions were prepared using a mixture of Capryol^TM^90, Transcutol®HP, Tween 80 and deionized water, with the mean particle being 10.4 nm for oral administration and 10.7 nm for intravenous injection. Pharmacokinetic study revealed that the absolute bioavailability of carotenoids in microemulsions and dispersion was 0.45% and 0.11%, respectively, while a much higher value of 6.25% and 1.57% were shown for lutein, demonstrating 4-fold enhancement in bioavailability upon incorporation of *R. nasutus* carotenoids into a microemulsion system.

Carotenoids, a group of lipid-soluble compounds with color ranging from yellow to red, can be divided into carotenes and xanthophylls, with the former containing only hydrocarbons and the latter being oxygenated derivatives^1^. Several epidemiological studies have shown that diets rich in carotenoid-containing foods are associated with reduced risk of certain types of chronic diseases such as cancer, cardiovascular disease, age-related macular degeneration and cataracts^2^,^3^,^4^,^5^,^6^. However, due to presence of long-chain conjugated double bonds, carotenoids are susceptible to degradations when exposed to oxygen, heat, light and acid, which in turn result in a low bioavailability *in vivo*^7^,^8^. For instance, O’Neill and Thurnham^9^ compared absorption efficiency of dietary supplements β -carotene (40 mg), lycopene (38 mg), and lutein (31.2 mg) in human, and reported that only 1.4 mg (3.5%), 1 mg (2.6%) and 0.8 mg (2.6%) were absorbed, respectively. Nevertheless, through development of a microemulsion or nanoemulsion technique for encapsulation of unstable carotenoids to enhance stability and bioavailability *in vivo* is feasible^8^. Compared to single intake of high-dose carotenoids, the multiple intakes of low-dose carotenoids were shown to provide a higher absorption efficiency^10^. In addition, through treatments of heat, mechanical force or enzyme, carotenoids in food matrix could be released faster to enhance bioavailability *in vivo*^11^. After release, the enzymatic hydrolysis of triglycerides by lipase for subsequent interaction with biliary salts for micelle formation is necessary, followed by transport to enterocyte membrane by simple diffusion or SR-B1 mediated transport, and chylomicron assembly for circulation to liver via lymphatic system^11^,^12^. In a previous study we demonstrated that through preparation of lycopene micelle and lycopene chylomicron, the absolute bioavailability of lycopene could be enhanced greatly, with the latter being higher than the former^8^.

*Rhinacanthus nasutus* (L.) Kurz (*R. nasutus*) is a well-known Chinese medicinal herb widely grown in Asian countries such as Taiwan and China, and often sold as “healthy beverage” on the market^13^. Several vital biological activities including anti-cancer^14^, anti-bacteria^15^ and anti-inflammation^16^ for consumption of *R. nasutus* have been well documented, which can be attributed to presence of various functional compounds like carotenoids, flavonoids, phenolic acids, chlorophylls, and napthoquinones^13^,^17^,^18^,^19^. Of various functional compounds, the carotenoid composition in *R. nasutus* has been thoroughly studied by Kao *et al.*^18^ and reported a total amount of 2195 μ g/g and 1576 μ g/g in freeze-dried and hot-air-dried samples, respectively. More specifically, all-trans forms of α -carotene, β -carotene, β -cryptoxanthin, lutein, neoxanthin and violaxanthin as well as their cis isomers were shown to be present, with all-trans-β -carotene dominating followed by all-trans-lutein and all-trans-α -carotene^18^. Regarding their biological significance, α -carotene and β -carotene are vitamin A precursors, while lutein has been demonstrated to be closely associated with prevention of age-related macular degeneration^12^,^20^. Thus, carotenoid-rich *R. nasutus* was chosen as a natural source for isolation and preparation of carotenoid microemulsion. By incorporating the carotenoid extract from *R. nasutus* into a microemulsion system, the bioavailability could be enhanced thereby reducing the dose substantially. Also, the unstable nature of carotenoids could be remedied. Most importantly, the bioavailability could be greatly enhanced by modifying the overall formula of *R. nasutus* healthy drink through incorporation of *R. nasutus*-derived carotenoid microemulsion.

Microemulsion belongs to a transparent or semi-transparent and thermodynamically stable emulsion, which can be formed by two immiscible liquids, oil and water, in the presence of surfactant or co-surfactant^21^. Due to the presence of extremely small droplets (<100 nm) in microemulsion, the phase separation phenomenon will not occur even after long-time storage. Though nanoemulsion systems are kinetically stable, they are not thermodynamically stable as microemulsions^21^. As only a few reported studies deal with bioavailability of carotenoid microemulsion and no information is available on bioavailability of *R. nasutus*-derived carotenoid microemulsion, the objectives of this study were to develop an open-column chromatographic method for isolation and preparation of carotenoids from *R. nasutus*. Then the various carotenoids were identified and quantified by HPLC-MS, followed by preparation of carotenoid microemulsion for oral bioavailability determination of both lutein and carotenoid using rats as an animal model.

## Results and discussion

### HPLC analysis of carotenoids

**Figure 1:**
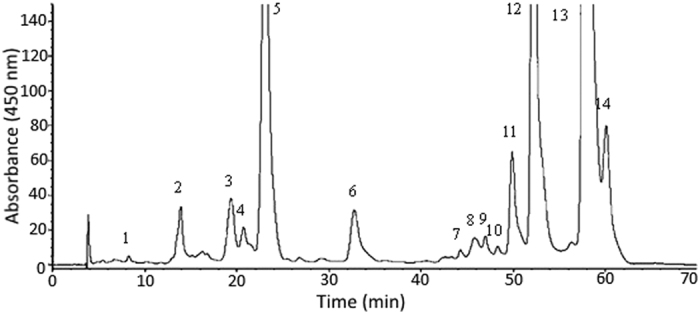
HPLC chromatogram of carotenoids prepared from *R. nasutus* extract by column chromatography. The identification of peaks is the same as shown in Table 1.

**Figure 2:**
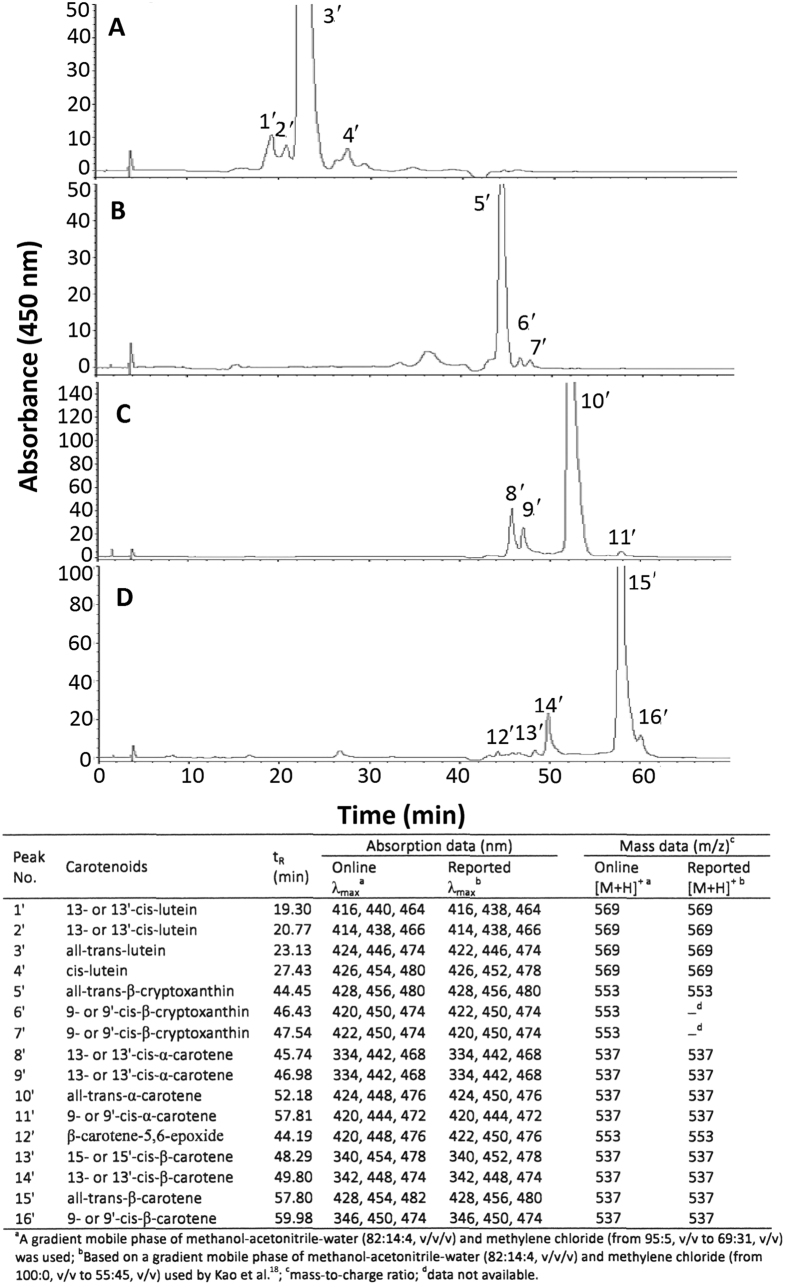
HPLC chromatograms along with absorption and mass spectra data for standards of all-trans forms of lutein (**A**), β -cryptoxanthin (**B**), α -carotene (**C**) and β -carotene (**D**) after illumination at 25 °C for varied time length.

Figure 1 shows HPLC chromatogram of carotenoids prepared from *R. nasutus* extract by open-column chromatography. A total of 14 carotenoids including internal standard β -apo-8′-carotenal were resolved within 62 min, with the retention factor (k) ranging from 1.16–14.73, separation factor (α ) from 1.03–2.27, and peak purity from 91.5–99.9% (Table 1), implying a proper solvent strength and selectivity of mobile phase to sample components was controlled. It has been well established that the k value should be controlled between 1–10 or 1–20 and α values higher than 1 to attain a satisfactory separation^22^. Nevertheless, several carotenoid peaks were partially overlapped in Fig. 1 and the complete resolution of all-trans forms of carotenoids and their geometrical isomers (cis-forms) has been difficult even with a C30 column, which may be due to highly complex nature of functional compounds present in herbs like *R. nasutus*. Furthermore, due to unavailability of cis-forms standards of carotenoids, the identification of cis-carotenoids by mass spectrometer is impossible. To overcome this problem, a method of photoisomerization of all-trans carotenoids was adopted for further identification of cis-carotenoids^18^. Figure 2 shows HPLC chromatograms of standards of all-trans forms of lutein (A), β -cryptoxanthin (B), α -carotene (C) and β -carotene (D) after illumination at 25 °C for varied length of time. A total of three cis isomers of lutein (cis-lutein and 13- or 13′-cis-lutein), two cis isomers of β -cryptoxanthin (9- and 9′ -cis-β -cryptoxanthin), three cis isomers of α -carotene (9- or 9′-cis-α -carotene and 13- or 13′-cis-α -carotene), three cis isomers of β -carotene (9- or 9′-cis-β -carotene, 13- or 13′ -cis-β -carotene and 15- or 15′-cis-β -carotene), as well as β -carotene-5,6-epoxide were identified based on absorption spectra and mass spectra characteristics (Fig. 2). In addition, neoxanthin and violaxanthin prepared from spinach by thin-layer chromatography (TLC) was also used for identification (See Supplementary Materials)^18^. On the basis of the identification and quantitation criteria as described in the method section, all-trans-β -carotene was found to be present in the largest amount (144 μ g/mL) in *R. nasutus* extract, followed by all-trans-lutein (50.3 μ g/mL), all-trans-α -carotene (49.2 μ g/mL), cis isomers of β -carotene (35.1 μ g/mL), cis isomers of lutein (8.94 μ g/mL), all-trans-violaxanthin (8.26 μ g/mL), cis isomers of α -carotene (5.39 μ g/mL), all-trans-β -cryptoxanthin (1.99 μ g/mL), and all-trans-neoxanthin (1.03 μ g/mL) (Table 1). However, no cis isomers of β -cryptoxanthin, neoxanthin and violaxanthin were detected, which should be caused by presence of their corresponding trans carotenoids in small amount.

### Characteristics of carotenoid microemulsion

**Figure 3:**
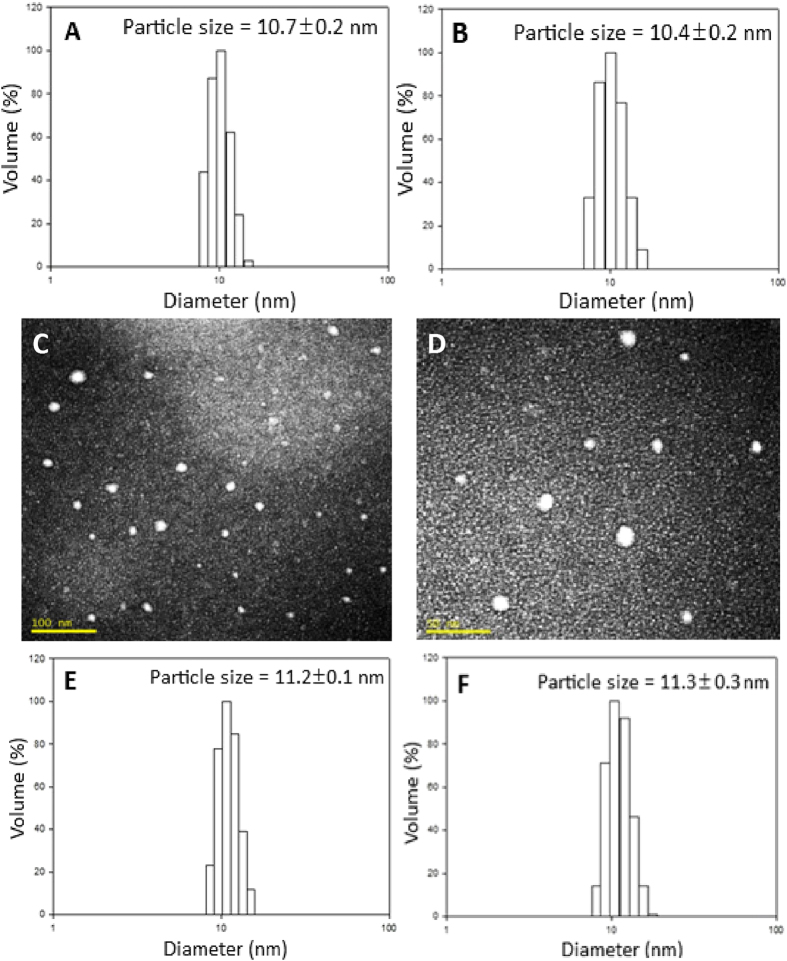
Characterization of carotenoid microemulsion with particle size distribution of microemulsion used for intravenous injection (**A**) and oral administration (**B**), TEM images captured at two different magnifications (**C**,**D**) and stability data for microemulsion used for intravenous injection (**E**) and oral administration (**F**) after storage at 25 °C for 90 days.

Figure 3A,B show particle size distribution of carotenoid microemulsion for intravenous (i.v.) injection (A) and oral administration (B) as determined by DLS, which equaled 10.7 nm and 10.4 nm, respectively. This microemulsion was successfully prepared based on a study by Zhang *et al.*^23^, reporting that Capryol^TM^ 90 is a suitable oil-soluble solvent for curcumin and Transcutol® HP is an appropriate water-soluble co-surfactant. Furthermore, Capryol^TM^ 90 is propylene glycol monocaprylate used in self-emulsifying systems to obtain fine dispersion for drug delivery, while Transcutol® HP is highly purified diethylene glycol monoethyl ether widely used for solubilizing poorly water-soluble drugs. Likewise, Tween 80, also known as polysorbate 80, is a nonionic surfactant and emulsifier derived from polyethoxylated sobitan and oleic acid. After various studies by following this approach, a carotenoid microemulsion was prepared by evaporating carotenoid extract to dryness under nitrogen, followed by adding 3% Capryol^TM^ 90 to dissolve the residue, 5% Transcutol® HP, 20% Tween 80, and 72% distilled water for complete homogenization, and sonicating for 90 min. This red transparent carotenoid microemulsion at a dose of 20 mg/kg body weight (bw) was used for oral administration of animal experiment. However, for i.v. injection, the carotenoid microemulsion at a dose of 2 mg/kg bw was prepared by mixing 2% Capryol^TM^ 90, 4% Transcutol® HP, 10% Tween 80 and 84% distilled water for complete homogenization and subsequent sonication. The difference in microemulsion composition is due to only one-tenth of carotenoid dose required for i.v. than that for oral administration^8^. Figure 3C,D show the TEM images of carotenoid microemulsion, indicating all the nanoparticles were well dispersed and possessed spherical shape with the average diameter being 12.1 nm, which is similar to that determined by DLS. A high encapsulation efficiency of 98.6% was obtained for carotenoid microemulsion, which is relatively higher than reported for several carotenoid nanosystems. For example, Gupta and Ghosh^24^ obtained only 79.6% encapsulation efficiency for β -carotene nanocapsules composed of ester-type plant sterols, flaxseed oil, calcium caseinate, sodium alginate and water, while a higher value of 89% was reported for carotenoid nanoparticles prepared using chitosan, Tween 80 and tripolyphosphate^25^. In another study, Xia *et al.*^26^ prepared carotenoid nanoliposome composed of cholesterol, Q10 coenzyme, Tween 80 and phosphate buffer and found that the encapsulation efficiency ranged from 90–95%. For the storage stability study, only a minor change in particle size of both microemulsions for i.v. and oral administration was observed, as evident by 11.2 nm and 11.3 nm nanoemulsion size even after a 90-day storage period, respectively (Fig. 3). The complete stability data at different intermittent storage times (15, 30, 45, 60, 75 and 90 days) can be found in the supplementary materials (Figure S3 and S4).

### HPLC analysis of carotenoids in rat serum

**Figure 4:**
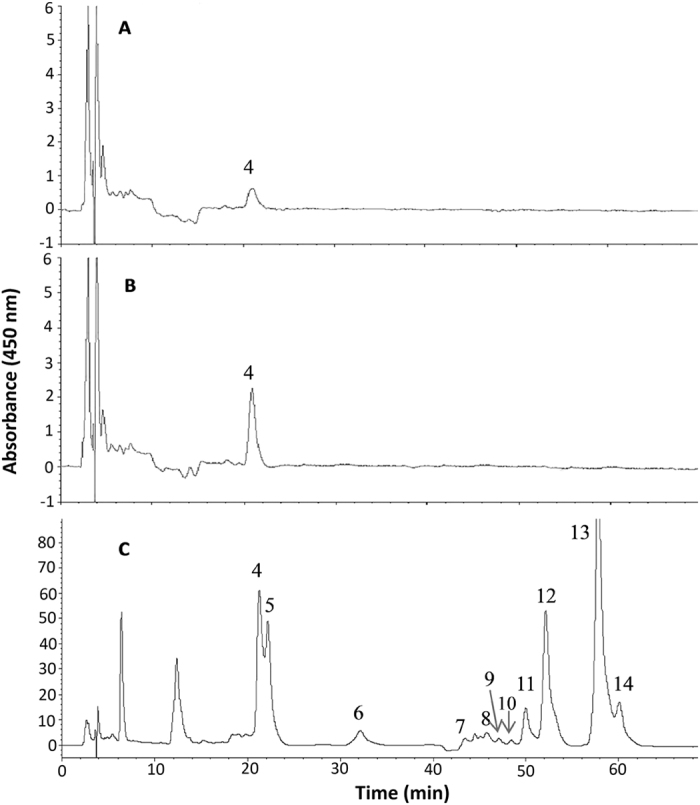
HPLC chromatograms for carotenoids in rat serum collected 4 h after oral administration of carotenoid dispersion (**A**) and carotenoid microemulsion (**B**) as well as 2 min after intravenous injection of carotenoid microemulsion (**C**). The identification of peaks is the same as shown in Table 1.

Figure 4 shows HPLC chromatograms of carotenoids in rat serum after oral administration of carotenoid dispersion (non-nano carotenoids) for 4 h (A), carotenoid microemulsions for 4 h (B) and i.v. injection of carotenoid microemulsions for 2 min (C). Only one carotenoid, 13- or 13′-cis-lutein (0.35 μ g/mL), was detected in rat serum for oral administration, which can be attributed to the instability nature of carotenoids such as neoxanthin and violaxanthin. Under acidic condition, the former could be converted to neochrome, while the latter converted to luteoxanthin or auroxanthin, accompanied by a color change from yellow to green or blue^18^. Also, it has been well documented that all-trans carotenoids can undergo degradation or convert to their corresponding cis isomers *in vivo*^27^,^28^. One of the major carotenoids in *R. nasutus*, all-trans-β -carotene remained undetected in rat serum for oral administration, which can be due to conversion to vitamin A. Accordingly, for oral administration, the conversion efficiency of all-trans-β -carotene to vitamin A in rats was 100%, but only 28% in humans^2^. Conversely, after i.v. injection for 2 min, a total of 10 carotenoids were detected, in which all-trans-β -carotene and its cis-isomers constituted the largest amount (26.0 μ g/mL), followed by all-trans-lutein and its cis isomers (14.0 μ g/mL), all-trans-α -carotene and its cis isomers (11.4 μ g/mL), and all-trans-β -cryptoxanthin (1.4 μ g/mL) (Fig. 4C). It is worth pointing out that the method validation of HPLC analysis of carotenoids in rat serum was not performed as it was carried out in our previous study^27^.

### Pharmacokinetic study

**Figure 5:**
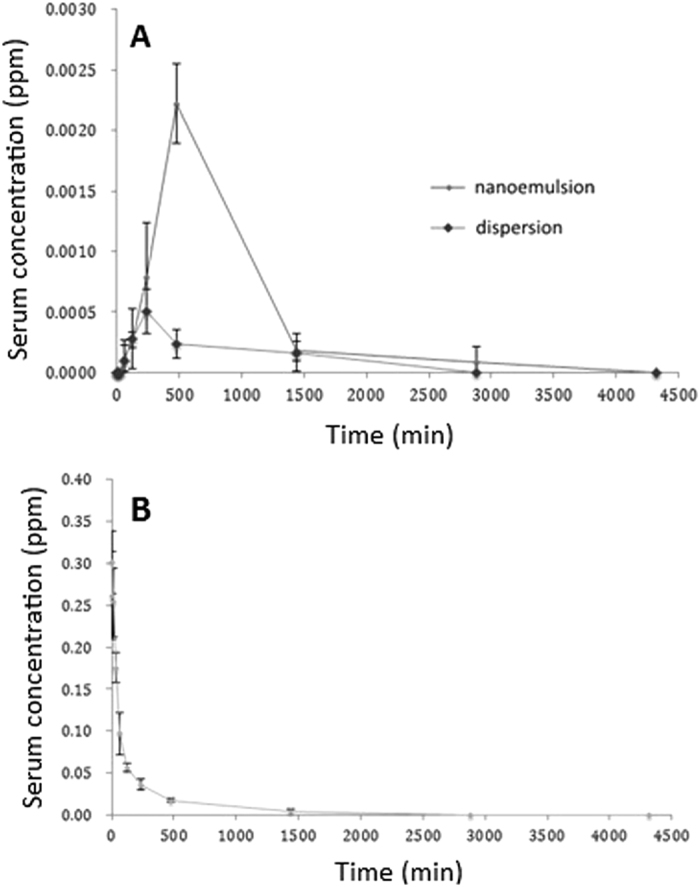
The concentration-time profile for carotenoids in rat serum after oral administration of carotenoid microemulsion and dispersion at 20 mg/kg bw (**A**) as well as intravenous injection at 2 mg/kg bw (**B**).

Figure 5A shows the concentration-time profile of carotenoids in rat serum after oral administration of carotenoid microemulsion and carotenoid dispersion at 20 mg/kg bw for varied length of time. No carotenoids were detected for both treatments after oral administration for 2, 5, 10 and 30 min. However, carotenoids remained undetected in rat serum until 1 h, then reached a plateau in 4 h for carotenoid dispersion and in 8 h for carotenoid microemulsion. Then the carotenoid level showed a declined trend thereafter and no carotenoids were detected in 48 h for carotenoid dispersion, but only a minor amount of carotenoids detected for carotenoid microemulsion in 48 h. Comparatively, under the same time length, a higher level of carotenoids in rat serum was shown for the microemulsion treatment than for the dispersion treatment, indicating a better absorption of the former.

Figure 5B shows the concentration-time profile of carotenoids in rat serum after i.v. injection of carotenoid microemulsion at 2 mg/kg bw for varied time length. A maximum carotenoid level was shown in rat serum at the initial time point (zero point), but followed a declined tendency thereafter, and no carotenoids were detected in 48 h. As mentioned before, in our study olive oil was used to dissolve carotenoids prepared by open-column chromatography to form dispersion, which makes it difficult for i.v. injection because of high viscosity. Thus, instead, for i.v. injection, only the treatment of microemulsion was used for pharmacokinetic study.

Table 2 shows pharmacokinetic parameters of carotenoids in rat serum after oral administration of carotenoid microemulsion and carotenoid dispersion at 20 mg/kg bw. The AUC for both microemulsion and dispersion treatments were 1.83 ±  1.02 and 0.46 ±  0.29 min μ g/mL, respectively, while the C_max_ were 2.20 ±  1.20 and 0.50 ±  0.20 ng/mL. Apparently the microemulsion treatment showed a significantly higher AUC and C_max_ than that of dispersion treatment. In addition, a much higher t_1/2_ was found for the microemulsion treatment than for the dispersion treatment, which amounted to 1520 ±  731 min and 1167 ±  699 min, respectively, implying a longer blood circulation time of the former. It is worth pointing out that the longer blood circulation time is a vital biomarker for enhanced bioavailability. All in all, the oral bioavailability of carotenoid microemulsion and carotenoid dispersion was calculated to be 0.45% and 0.11%, respectively, demonstrating a higher absorption efficiency of the former. As mentioned before, the extremely low bioavailability of total carotenoids in rats can be caused by degradation and isomerization as well as conversion of provitamin A carotenoids to vitamin A.

The pharmacokinetic parameters of lutein in rat serum after oral administration carotenoid microemulsion and carotenoid dispersion at 20 mg/kg bw is also shown in Table 2. The AUC of lutein for both microemulsion and dispersion treatments were 1.83 ±  1.02 and 0.46 ±  0.29 min μ g/mL, respectively, while the C_max_ of lutein were 2.20 ±  1.20 and 0.50 ±  0.20 ng/mL. Also, a much higher t_1/2_ and T_max_ was found for lutein in the carotenoid microemulsion treatment than that for the dispersion treatment. By comparison, the pharmacokinetic parameters of lutein were the same as carotenoids for both dispersion and microemulsion by oral administration, mainly because only lutein was detected after oral administration. The oral bioavailability of lutein was determined to be 6.25% and 1.57% for the carotenoid microemulsion treatment and carotenoid dispersion treatment, respectively. Comparatively, in our experiment lutein showed a much higher bioavailability than the other carotenoids in rats, which should be due to a higher stability of the former. In addition, most importantly, carotenoids in *R. nasutus* extract contain a high amount of α -carotene and β -carotene (Table 1), both of which can be converted to vitamin A in rats leading to a further decline in carotenoid bioavailability. Also, as mentioned above, both neoxanthin and violaxanthin were susceptible to degradations when exposed to acid in the stomach.

The absolute bioavailability of carotenoid microemulsion or nanoemulsion can be affected by many factors such as shape, size, dose, encapsulation efficiency, emulsion characteristics, formulation, etc. For example, in a recent study Chen *et al.*^8^ reported that a high dose of lycopene may cause saturation of lycopene absorption in rats. Instead, a high efficiency in lycopene absorption was shown at low dose (10 or 13 mg per day) in a human clinical trial, probably due to the presence of intestinal-binding protein to facilitate lycopene absorption^29^,^30^. In another study Salvia-Trujillo *et al.*^31^ prepared β -carotene emulsions of different size (23, 0.38 and 0.21 μ m) and reported that the smaller the size, the higher the absorption efficiency *in vitro*. Likewise, Wang *et al.*^32^, prepared β -carotene emulsion composed of soybean oil and decaglycerol monolaurate and demonstrated that the smaller the size, the higher the bioavailability *in vitro*. Compared to the other carotenoids, lutein was shown to possess a higher absorption efficiency, which can be associated with its stability and polar nature^33^. In addition to carotenoids, Hatanaka *et al.*^34^ prepared Q10 nanoemulsion composed of surfactant, lecithin, glycerol, and water with the average size being 60 nm, and performed the pharmacokinetic experiments in rats. The outcome showed that the Q10 nanoemulsion resulted in a 1.7-fold higher AUC and C_max_ than the non-nano treatment. Similarly, Kotyla *et al.*^35^ prepared vitamin E emulsion and nanoemulsion composed of canola oil and polysorbate 80 with size being 2788 nm and 65 nm, respectively, and reported that the vitamin E concentration in rat blood was much higher for nanoemulsion than for emulsion, demonstrating again the smaller the size, the better the absorption. Interestingly, in a recent study Chen *et al.*^8^ prepared lycopene micelle and lycopene chylomicron with tomato extract as raw material with the size being 7.5 nm and 131.5 nm, respectively, based on TEM analysis, and the absolute bioavailability was determined to be 6.8% for the former and 9.5% for the latter. It was postulated that both size and shape should be taken into account for bioavailability determination as a higher bioavailability was shown for lycopene chylomicron with a thicker outer layer^8^. Moreover, for i.v. injection in our present study, a much smaller size of carotenoid microemulsion (12.1 nm) may evade the body’s reticuloendothelial system and penetrate into small capillaries more readily and thus the blood circulation time can be greatly extended^8^. Another important factor in blood circulation time extension can be attributed to a large surface area of carotenoid microemulsion which can facilitate solubilization of lipophilic carotenoids to enhance absorption^21^. Also, the possibility of renal excretion of carotenoid microoemulsion can be excluded as the cut-off size for renal filtration was reported to be 5.5 nm^36^. Conversely, a short blood-circulation time can be attributed to particles with diameter >200 nm caused by separation by mechanical filtration in the spleen and then removal by the phagocyte^37^. Thus, it is possible for the i.v. injection that the microemulsion will persist in the blood depending upon size, stability and time length. But for oral administration, the microemulsion will mix with bile salts and then change when they pass through the epithelium cells.

In conclusion, a preparative column chromatographic method was developed to separate carotenoids from *R. nasutus* extract with magnesium oxide-diatomaceous earth (1:3, w/w) as adsorbent and ethyl acetate as eluent. An HPLC gradient solvent system composed of methanol/methylene chloride/water (82:14:4) (A) and methylene chloride (B) could resolve 14 carotenoids including internal standard β -apo-8′ -carotenal within 62 min with flow rate at 1.0 mL/min and detection at 450 nm. A carotenoid microemulsion composed of Capryol^TM^ 90, Transcuton® HP, Tween 80 and distilled water was successfully prepared with the average size being 10.4 nm and 10.7 nm for oral administration and i.v. injection, respectively. Also, the microemulsion showed a high stability over a 90-day storage period. The absolute bioavailability of carotenoid in microemulsion and dispersion was 0.45% and 0.11%, respectively. However, the absolute bioavailability of lutein in microemulsion and dispersion was much higher than carotenoid, which amounted to 6.25% and 1.57%, respectively.

## Materials and methods

### Materials

A total of 6 kg fresh *Rhinacanthus nasutus* (L.) Kurz (*R. nasutus*) was purchased from a local Chinese drug store located in Wan-Hua district, Taipei city. After cleaning and freeze-drying to moisture content <10%, a total of about 500 g *R. nasutus* was obtained and placed into separate plastic bags with 25 g each and sealed under vacuum for storage at −20 °C prior to use.

Carotenoid standards including all-trans forms of zeaxanthin, β -cryptoxanthin, α -carotene and β -carotene were purchased from Sigma (St. Louis, MO, USA), while all-trans-lutein was from Fluka Chemical Co. (Buchs, Switzerland) and all-trans-neoxanthin from Chromadex Co (CA, USA). Internal standard all-trans-β -apo-8′ -carotenal was also from Fluka Chemical Co. Both neoxanthin and violaxanthin standards were prepared from spinach by TLC using a method as described by Kao *et al.*^18^. The HPLC-grade solvents including methanol, ethanol, acetone, ethyl acetate, acetonitrile, toluene and methylene chloride were obtained from Lab-Scan Co. (Gliwice, Poland). The analytical grade solvent n-hexane was from Grand Chemical Co. (Taipei, Taiwan). Deionized water was made using a Milli-Q water purification system from Millipore Co. (Bedford, MA, USA). Magnesium oxide, potassium hydroxide and potassium phosphate were from Sigma, while diatomaceous earth was from J.T. Baker Co. (Phillipsburg, NJ, USA). Both Capryol^TM^ 90 and Transcutol® HP were from Gattefosse Co. (Saint-Priest, France). Tween 80 was from Yi-Pa Co. (Taipei, Taiwan).

### Instrumentation

The HPLC-MS system (The Agilent Technologies Co. 1200 series) is composed of a G1379B degasser, a G1312B pump, an auto sample injector (G1329B), a column temperature controller (G1316B), a photodiode-array detector (G1315C), and a 6130 quadrupole mass spectrometer with multi-mode ion source (ESI and APCI). The polymeric C_30_ reversed-phase column (250 ×  4.6 mm ID, 5 μ m particle size) and guard column (6 ×  4.6 mm ID) was from YMC Co. (Milford, MA, USA). The spectrophotometer (DU 640) was from Beckman Co. (Fullerton, CA, USA). The Eyela N-1 rotary evaporator was from Tokyo, Japan. The freeze-dryer was from Chin-Ming Co. (Taipei, Taiwan). The sonicator (DC400H) was from Taipei, Taiwan. The high-speed centrifuge (Sorvall RC5C) was from DuPont Co. (Wilmington, DL, USA). The micro centrifuge (Fresco 21) was from Thermo Co. (USA). The dynamic light scattering (DLS) instrument was from Brookhaven Instrument Co. (Holtsville, NY, USA). The transmission electron microscopy (TEM) (JEM-1400) was from JEOL Co. (Tokyo, Japan).

### Extraction of carotenoids

A method based on Inbaraj *et al.*^38^ was used to extract carotenoids from *R. nasutus* samples. Initially, a 10-g powdered *R. nasutus* sample was mixed with 80 mL of hexane/ethanol/acetone/toluene (10:6:7:7, v/v/v/v) in a flask, after which the solution was shaken at room temperature for 1 h, followed by adding 80 mL hexane, shaking again for 10 min, and adding 30 mL anhydrous sodium sulfate (10%) for partition. The upper layer containing carotenoid was collected, while the lower layer was added with 30 mL hexane for repeated extraction until colorless. All the supernatants were pooled, evaporated to dryness and dissolved in 10-mL hexane to obtain crude carotenoid extract. After filtration through a 0.22-μ m membrane filter, a 20-μ L sample was injected into HPLC-MS for qualitative and quantitative analyses of carotenoids.

### Preparation of carotenoids by open-column chromatography

A method based on Loh *et al.*^39^ was modified to isolate and prepare carotenoids from *R. nasutus* samples. A 3-mL carotenoid extract was poured into a glass column (400 ×  42 mm ID) containing a mixture (52 g) of magnesium oxide and diatomaceous earth (1:3, w/w), which was pre-activated with 500-mL hexane. Then anhydrous sodium sulfate was added above the adsorbent to form about 1-cm layer. Next, 25 mL of hexane (100%) was added for equilibrium, followed by adding 250 mL of ethyl acetate (100%) to elute carotenoids. The eluate was then evaporated to dryness, dissolved in 5-mL methanol/methylene chloride (3:7, v/v) and filtered through a 0.22-μ m membrane filter for HPLC analysis. The isolation of carotenoid fraction in an open-column is shown in the supplementary material (Figure S1).

### HPLC analysis of carotenoids in *R. nasutus* extract

An HPLC method based on Kao *et al.*^18^ was modified to separate various carotenoids in *R. nasutus* extract by using a Waters YMC C30 column (250 ×  4.6 mm ID, 5 μ m particle size) with flow rate at 0.8 mL/min, detection at 450 nm and a mobile phase of methanol-acetonitrile-water (82:14:4, v/v/v) (A) and methylene chloride (100%) (B) with the following gradient elution: 95% A and 5% B initially, maintained for 5 min, decreased to 90% A at 8 min, 86% A at 10 min, maintained for 26 min, 70% A at 38 min, maintained for 12 min, 69% A at 52 min, maintained for 16 min, and returned to original ratio at 70 min. The various carotenoids in *R. nasutus* extract were identified by comparing retention times, absorption spectra and mass spectra of unknown peaks with those of reference standards. A single quadrupole mass spectrometer with APCI mode was used for detection with scanning range 400–1200 m/z, drying gas flow 7 L/min, nebulizer pressure 10 psi, dry gas temperature 330 °C, vaporizer temperature 230 °C, capillary voltage 2000 V, charging voltage 2000 V, corona current 4 μ A and fragmentor voltage 200 V. In addition, a photoisomerization method of carotenoid standards including all-trans forms of lutein, β -carotene, β -cryptoxanthin and zeaxanthin was used for further identification of cis-isomers of carotenoids (See Supplementary Materials)^18^. For quantitation, an internal standard β -apo-8′ -carotenal at a concentration of 10 μ g/mL was mixed with each standard solution. Various concentrations of carotenoid standards including all-trans-β -carotene (1–200 μ g/mL), all-trans-zeaxanthin (2–20 μ g/mL), all-trans-β -cryptoxanthin (1–20 μ g/mL), all-trans-α -carotene (2–80 μ g/mL), all-trans-lutein (2–80 μ g/mL and 0.1–1.5 μ g/mL), all trans-neoxanthin (1–10 μ g/mL) and violaxanthin (3.09–77.25 μ g/mL) were prepared and the standard curve of each carotenoid was drawn by plotting concentration ratio against area ratio, with the linear regression equation and correlation coefficient (R) being obtained by an EXCEL software system. For preparation of neoxanthin and violaxanthin standards, neoxanthin and violaxanthin isolated from spinach extract by TLC were quantified by spectrophotometric analysis at 439 nm and 443 nm, respectively, and were found to be 29.4 μ g/mL and 309 μ g/mL (See Figure S2 in Supplementary Materials)^18^. The linear regression equations of all-trans forms of neoxanthin, violaxanthin, lutein, β -cryptoxanthin, α -carotene and β -carotene were y =  2.0589x +  0.0092, y =  1.5064x−0.1768, y =  0.5524x −  0.0775, y =  2.1876x +  0.0626, y =  0.5075x +  0.0948 and y =  0.5935x +  0.2004, respectively, with R being all higher than 0.99.

### Preparation of carotenoid microemulsion

A method based on Zhang *et al.*^23^ and Chen *et al.*^8^ was modified to prepare carotenoid microemulsion from *R. nasutus* extract. For intravenous injection (2 mg/kg bw), an appropriate amount of carotenoid extract was evaporated to dryness, followed by adding 0.2 g of Capryol^TM^ 90, 0.4 g of Transcutol® HP, 1.0 g of Tween 80 and 8.4 g of distilled water. After mixing thoroughly, the mixture was sonicated for 90 min to obtain a 10-mL carotenoid microemulsion. For oral administration (20 mg/kg bw), a suitable amount of carotenoid extract was evaporated to dryness, followed by adding 0.3 g of Capryol^TM^ 90, 0.5 g of Transcutol® HP, 2.0 g of Tween 80, and 7.2 g of distilled water. After mixing thoroughly, the mixture was sonicated for 90 min to obtain a 10-mL carotenoid microemulsion.

### Particle size determination

The particle size distribution of carotenoid microemulsion was determined by DLS using a method as described by Chen *et al.*^8^. In the beginning the KH_2_PO_4_ (potassium dihydrogen phosphate) buffer solution was prepared by dissolving 1.7 g of KH_2_PO_4_ in 200-mL deionized water, followed by adjusting pH to 5.5 with 0.1 M potassium hydroxide, and diluting to 250 mL with deionized water. Then 100-μ L of carotenoid microemulsion was collected and diluted to 5 mL with KH_2_PO_4_ buffer solution, after which the microemulsion was filtered through a 0.2-μ m membrane filter and transferred to a polystyrene tube for determination of particle size distribution by DLS at 25 °C and the data were analyzed by a BIC particle sizing 90 plus software system. In addition to DLS, TEM was also used to determine particle size and shape based on a method by Chang and Chen^40^. Prior to TEM analysis, carotenoid microemulsion was diluted 50 times with deionized water, after which 20 μ L was collected and dropped onto a carbon coated 74 μ m copper grid for 30 s, followed by removing excessive sample with a glass filter paper, negative staining for 30 s with 20 μ L of 2% PTA, removing excessive stain again with a glass filter paper and drying in a dessicator for overnight. Then the TEM image was recorded by enlarging sample 3 ×  10^5^ times under 120 kV.

### Encapsulation efficiency

The encapsulation efficiency was determined based on a method reported by Chang and Chen^40^ by mixing 200 μ L of carotenoid microemulsion with 200 μ L of 25 mM potassium dihydrogen phosphate buffer solution (pH 5.5) and poured into a centrifuge tube equipped with a dialysis membrane (molecular weight cut-off 3 kDa) for centrifugation at 12,000 rpm for 20 min. The solution passed through the membrane was dried, followed by dissolving the residue in 100 μ L methylene chloride, adding 100 μ L of 20 ppm internal standard β -apo-8′ -carotenal dissolved in methylene chloride and injecting 20 μ L into HPLC. Based on the amount of free carotenoid, the encapsulation efficiency can be calculated using the formula as shown below:


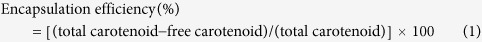


### Storage stability of microemulsion

Carotenoid microemulsion was stored at 25 °C for 3 months and sample was collected every 15 days for determination of particle size distribution by DLS and observation of phase separation by eye.

### Animal experiment

Male Sprague-Dawley rats with body weight 230–250 g were procured from BioLASCO (Taipei, Taiwan), after which these animals were transported to Fu Jen University Laboratory Animal Center. A prior approval for using male Sprague-Dawley rats for this study was obtained from Fu Jen University animal subjects review committee. These animals were housed in ventilation cages at an ambient temperature of 21 ±  2 °C and relative humidity of 55 ±  10% for 12 h under light and 12 h in the dark. All the rats were fed with a sterilized laboratory rodent diet 5010 (LabDiet Co., St. Louis, MO, USA) ad libitum. After the body weight of all the rats reached about 280 g (8-week old), rats were ready for experiments. Also, all the 18 rats were prohibited from feeding for 12 h prior to experiments. Most importantly, the methods involving animal experiments have been carried out with the approved guidelines^41^.

Three treatments were used: the first (6 rats) and the second (6 rats) received oral administration of carotenoid dispersion (in oil) and microemulsion, respectively, while the third (6 rats) received i.v. injection of carotenoid microemulsion. The treatment of carotenoid dispersion by i.v. injection was not carried out because of high viscosity. For oral administration, both carotenoid dispersion and microemulsion were fed to rats separately at a dose of 20 mg/kg based on carotenoid concentration. This dose was selected based on several trials. After oral administration for 2, 5, 10 and 30 min and 1, 2, 4, 8, 24, 48 and 72 h, 0.6 mL of blood was collected from the tail vein, followed by pouring into a heparin-rinsed tube, transferring to ice bath for 30 min, and centrifuging at 5000 rpm for 15 min (4 °C). Then the supernatant was collected for subsequent carotenoid extraction and HPLC analysis. For i.v. injection, carotenoid microemulsion was injected into the temporal vein of rats at a dose of 2 mg/kg, which was one-tenth that of oral administration. After injection for 2, 5, 10 and 30 min, and 1, 2, 4, 8, 24, 48 and 72 h, 0.5 mL of blood was collected from the tail vein, followed by pouring into a heparin-rinsed tube, transferring into ice bath for 30 min, and centrifuging at 5000 rpm for 15 min at 4 °C. Then the supernatant was collected for subsequent carotenoid extraction and HPLC analysis.

### HPLC analysis of carotenoids in serum

A method based on Hsu *et al.*^27^ was modified. Initially serum sample was poured into a 15-mL centrifuged tube, and 1 mL of ethanol solution containing 0.01% of ascorbic acid was added for protein precipitation and prevention of oxidation. Then 1 mL of ethyl acetate and 3 mL of hexane were added, after which the mixture was vortexed for 10 s and then shaken in a shaker for 10 min at 200 rpm. After centrifuging at 3000 rpm for 20 min at 4 °C, the supernatant was collected and 3-mL hexane was added to the lower layer 3 times for repeated extraction of carotenoids. All the supernatants were pooled, evaporated to dryness under N_2_, dissolved in 50-μ L methylene chloride containing internal standard parared (2 μ g/mL), filtered through a 0.22 μ m membrane filter, and 20-μ L was injected for HPLC analysis. The various carotenoids in serum samples were identified and quantified using the same approach as described above.

### Pharmacokinetic study

Pharmacokinetic study was performed using a WinNonlin software system (Pharsight Co., CA, USA) by a non-compartmental model with the data expressed as mean ±  standard deviation^8^. The area under the drug concentration-time curve (AUC) was used to determine the total amounts of carotenoids and lutein reaching systemic circulation. In addition, some other kinetic parameters such as T_max_, C_max_ and t_1/2_ were determined. The absolute availability of carotenoid and lutein was calculated using the following formula:


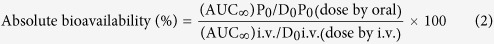


### Statistical analysis

All the experimental data were subjected to analysis of variance and Student’s paired t-test for significance in mean comparison at p <  0.05^42^.

## Additional Information

**How to cite this article**: Ho, N. H. *et al.* Utilization of Microemulsions from *Rhinacanthus nasutus* (L.) Kurz to Improve Carotenoid Bioavailability. *Sci. Rep.* **6**, 25426; doi: 10.1038/srep25426 (2016).

## Supplementary Material

Supplementary Information

## Figures and Tables

**Table 1:** Retention time (t_R_), retention factor (*k*), separation factor (α), peak purity (pp) and contents of various carotenoids in carotenoid fraction isolated from *R. nasutus* extract along with their absorption and mass spectra data for identification.

Peak No.	Carotenoid	t_R_ (min)	*k*	α	pp (%)	Absorption data (nm)						Mass data (m/z)^i^		Content (μ g/mL)
						Online λ_max_^f^			Reported λ_max_^h^			Online [M + H]^+^	Reported [M + H]^+^^h^	
1	all-trans-neoxanthin^a^	8.25	1.16	2.27 (1, 2)^e^	98.8	418	440	468	418	440	468	601	601	1.03 ± 0.01
2	all-trans-violaxanthin^a^	13.88	2.63	1.54 (2, 3)^e^	96.4	416	440	468	416	440	468	601	601	8.26 ± 0.74
3	13- or 13′ -cis-lutein^b^	19.35	4.07	1.09 (3, 4)^e^	95.6	416	440	464	416	438	464	569	569	5.59 ± 0.88
4	13- or 13′ -cis-lutein^b^	20.73	4.43	1.14 (4, 5)^e^	91.5	414	438	466	414	438	466	569	569	3.35 ± 0.78
5	all-trans-lutein^c^	23.07	5.04	1.50 (5,IS)^e^	99.3	424	446	474	422	446	474	569	569	50.3 ± 2.69
6	β -apo-8′ -carotenal (IS)^d^	32.74	7.57	1.40 (IS, 7)^e^	99.4	–g	464	–g	–g	464	–g	417	417	–g
7	all-trans-β -cryptoxanthin^c^	44.24	10.58	1.04 (7, 8)^e^	97.2	428	456	480	428	456	480	553	553	1.99 ± 0.91
8	13- or 13′ -cis-α -carotene^b^	45.77	10.98	1.03 (8, 9)^e^	97.7	334	442	468	334	442	468	537	537	2.95 ± 0.32
9	13- or 13′ -cis-α -carotene^b^	46.96	11.29	1.03 (9, 10)^e^	94.2	334	442	468	334	442	468	537	537	2.44 ± 0.06
10	15- or 15′ -cis-β -carotene^b^	48.31	11.65	1.04 (10, 11)^e^	98.6	340	454	478	340	452	478	537	537	2.70 ± 0.15
11	13- or 13′ -cis-β -carotene^b^	49.88	12.06	1.05 (11, 12)^e^	99.6	342	448	474	342	448	474	537	537	12.8 ± 0.92
12	all-trans-α -carotene^c^	52.17	12.66	1.12 (12, 13)^e^	99.7	424	448	476	424	450	476	537	537	49.2 ± 2.69
13	all-trans-β -carotene^c^	57.82	14.14	1.04 (13, 14)^e^	99.9	428	454	482	428	456	482	537	537	144 ± 7.78
14	9- or 9′ -cis-β -carotene^b^	60.07	14.73	1.04 (13, 14)^e^	99.6	346	450	474	340	450	476	537	537	19.6 ± 1.34

^a^Identification based on absorption and mass spectra of samples isolated from spinach by thin-layer chromatography.

^b^Identification based on absorption and mass spectra of HPLC chromatogram obtained for photoisomerized all-trans standards as shown in Fig. 2.

^c^Identification based on absorption and mass spectra of commercially obtained reference standards.

^d^Internal standard.

^e^Numbers in parentheses represent values between two neighboring peaks.

^f^A gradient mobile phase of methanol-acetonitrile-water (82:14:4, v/v/v) and methylene chloride (from 95:5, v/v to 69:31, v/v) was used.

^g^Data not available.

^h^Based on a gradient mobile phase of methanol-acetonitrile-water (82:14:4, v/v/v) and methylene chloride (from 100:0, v/v to 55:45, v/v) used by Kao *et al.*^18^

^i^m/z is mass-to-charge ratio.

**Table 2:** Pharmacokinetic parameters* of carotenoids and lutein in rat serum after oral administration of carotenoid microemulsion and dispersion at 2 and 20 mg/kg bw, respectively.

Parameters	Oral administration		Intravenous injection of microemulsion
	Dispersion in oil	Microemulsion	
Carotenoids			
T_max_ (min)^b^	240	480	–
C_max_ (ng/mL)^c^	0.50 ± 0.20	2.20 ± 1.20^a^	310.13 ± 30.14
t_1/2_ (min)^d^	1167.83 ± 699.74	1520.75 ± 731.25	215.17 ± 90.19
AUC (min μ g/mL)^e^	0.46 ± 0.29	1.83 ± 1.02^a^	40.80 ± 6.55
Oral bioavailability (%)	0.11 ± 0.07	0.45 ± 0.25^a^	–
Lutein			
T_max_ (min)^b^	240	480	–
C_max_ (ng/mL)^c^	0.50 ± 0.20	2.20 ± 1.20^a^	99.87 ± 11.45
t_1/2_ (min)^d^	1167.83 ± 699.74	1520.75 ± 731.25	15.73 ± 2.79
AUC (min μ g/mL)^e^	0.46 ± 0.29	1.83 ± 1.02^a^	2.95 ± 0.37
Oral bioavailability (%)	1.57 ± 1.00	6.25 ± 3.50^a^	–

^*^Data expressed as mean ±  standard deviation (n =  6 for each group).

^a^Significantly different (p <  0.05) data when compared with dispersion group as determined by Student’s t-test.

^b^Time to reach C_max_.

^c^Maximum serum concentration.

^d^Time to reach half concentration.

^e^Area under the concentration-time curve.
