# Supplementary material for: Utilization of Microemulsions from Rhinacanthus nasutus (L.) Kurz to Improve Carotenoid Bioavailability
Source: Sci Rep. 2016 May 6;6:25426. doi: 10.1038/srep25426 (PMC4858739; doi:10.1038/srep25426)
Supplement: Supplementary Information [file srep25426-s1.doc]

**Utilization of Microemulsions from *Rhinacanthus nasutus* (L.) Kurz to Improve Carotenoid Bioavailability**

**N.H. Ho, B. Stephen Inbaraj and B.H. Chen***

Department of Food Science, Fu Jen University, Taipei 242, Taiwan

*Corresponding author: Tel: +886-2-29053626, Fax: +886-2-29051215,

E-mail: 002622@mail.fju.edu.tw

_____________________________________________________________________

**Photoisomerization method for identification of cis-isomers**

Due to the presence of conjugated double bonds, carotenoids are very susceptible to isomerization upon exposure to light, oxygen, heat, acid and alkali. Accordingly, the cis-isomers in *R. nasutus* extract were identified based on a photoisomerization method reported by Kao et al.^6^ by generating cis-isomers through exposure of carotenoid standards to light. More elaborately, 1 mL of 100 mg/L all-trans-β-cryptoxanthin, all-trans-α-carotene and all-trans-β-carotene standards dissolved in methylene chloride were separately poured into transparent glass vials and irradiated with 2000-3000 lux under fluorescent tubes in an incubator at 25°C for 24 h. For illumination of all-trans-lutein, 1 mL of 100 mg/L standard dissolved in methylene chloride was mixed with 100 μL of iodine and illuminated for 1 h following the same procedure as described above. After illumination, 20 μL of each sample was injected into HPLC-DAD-MS for separation and identification.

**Preparation of neoxanthin and violaxanthin from spinach**

A method based on Kao et al.^6^ was used to prepare neoxanthin and violaxanthin standards from spinach. Initially, 25 g of spinach powder was mixed with with 450 mL of hexane and shaken for 1 h. Then, 90 mL of 40% methanolic potassium hydroxide was added for saponification for 16 h under nitrogen in a dark room, followed by adding 300 mL of n-hexane, shaking for 10 min, adding 150 mL of 10% anhydrous sodium sulfate solution, shaking again for 1 min and allowing to stand at room temperarure for separation into two layers. The supernatant containing the carotenoid extract was collected, evaporated to dryness and dissolved in 10 mL of n-hexane.

For TLC separation, initially the silica gel 60 F254 TLC plates (20cm x 20 cm, 0.5 mm thickness) were dried in a 100°C oven for 1 h for activation. Next, 150 mL of methanol-acetone-hexane (1:29:70, v/v/v) was poured into a developing tank and allowed for vapor saturation at room temperature for 30 min. By using a micropipette, 2 μL of spinach extract was spotted on 12 points at a distance of 3 cm from the bottom of TLC plate. Each point was loaded with extract for 15 times and dried, followed by placing the TLC plate in the tank for the solvent system to reach a distance of 18 cm from the top. Then, the first and second yellow spots were scratched with a spatula separately and dissolved in acetone. The same procedure was repeated with 20 TLC plates. After filtration, both neoxanthin and violaxanthin were evaporated to dryness and dissolved in ethanol for quantitative analysis using a spectrophotometer at 439 nm and 443 nm, respectively. According to Beer’s law and an extinction coefficient of 2243 and 2550, the amount of neoxanthin and violaxanthin was determined to be 29.4 μg/mL and 309 μg/mL, respectively.


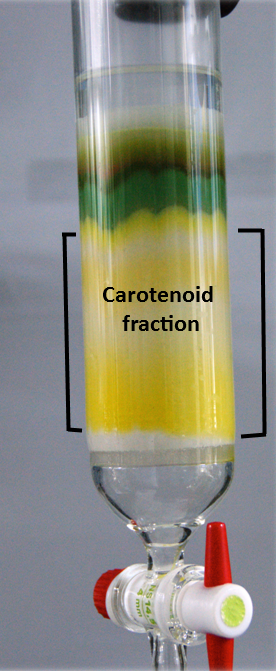


**Figure S1 – Open-column chromatographic isolation of carotenoids from *Rhinacanthus nasutus* using 250 mL ethyl acetate poured over 2 mL crude extract loaded on a column packed with 52 g of magnesium oxide-diatomaceous earth**

**(1:3, w/w)**


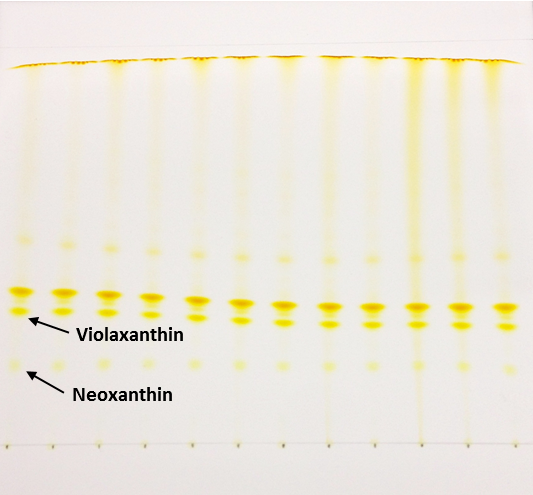


**Figure S2 – Thin-layer chromatographic isolation of violaxanthin and neoxanthin from spinach extract on a silica gel 60 F254 TLC plate using a solvent system of methanol-acetone-hexane (1:29:70, v/v/v)**


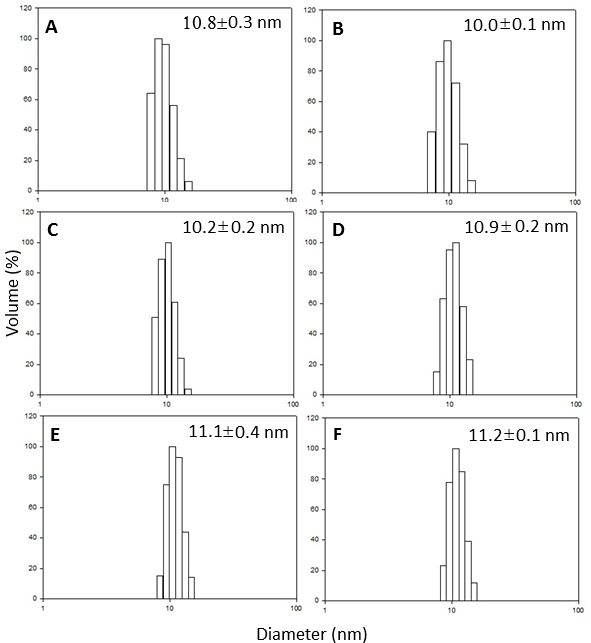


**Figure S3 – Stability data of carotenoid microemulsion used for intravenous injection showing minor change in particle size distribution upon storage at 25°C for 15 days (A), 30 days (B), 45 days (C), 60 days (D), 75 days (E) and 90 days (F).**


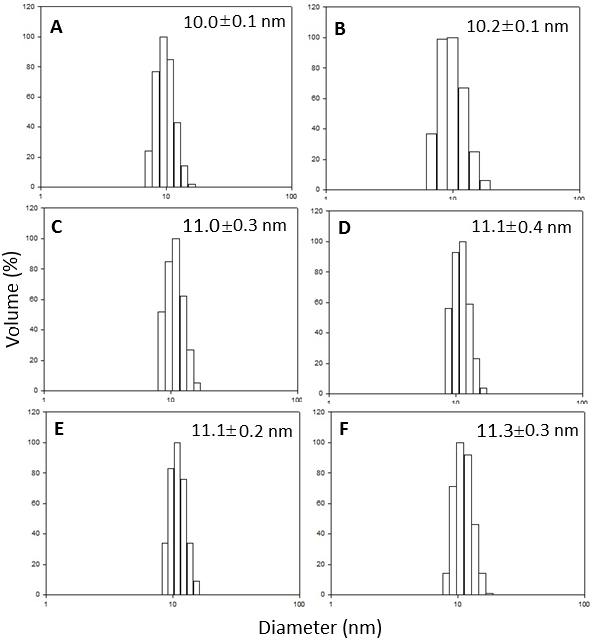


**Figure S4 – Stability data of carotenoid microemulsion used for oral administration showing minor change in particle size distribution upon storage at 25°C for 15 days (A), 30 days (B), 45 days (C), 60 days (D), 75 days (E) and 90 days (F).**
